# FAD104, a regulator of adipogenesis, is a novel suppressor of TGF-β–mediated EMT in cervical cancer cells

**DOI:** 10.1038/s41598-017-16555-3

**Published:** 2017-11-27

**Authors:** Motoharu Goto, Shigehiro Osada, Masayoshi Imagawa, Makoto Nishizuka

**Affiliations:** Department of Molecular Biology, Graduate School of Pharmaceutical Sciences, Nagoya City University, 3-1 Tanabe-dori, Mizuho-ku, Nagoya, Aichi 467-8603 Japan

## Abstract

Epithelial-to-mesenchymal transition (EMT) is a biological process in which epithelial cells translate into a mesenchymal phenotype with invasive capacities, contributing to tumour progression, metastasis, and the acquisition of chemotherapy resistance. To identify new therapeutic targets for cancers, it is important to clarify the molecular mechanism of induction of EMT. We have previously reported that *fad104*, a positive regulator of adipocyte differentiation, suppressed the invasion and metastasis of melanoma and breast cancer cells. In this study, we showed that FAD104 functions as a novel suppressor of transforming growth factor-β (TGF-β)–mediated EMT in cervical cancer cells. Expression of FAD104 is upregulated during TGF-β–mediated EMT in human cervical cancer HeLa cells. Reduction of *fad104* expression enhanced TGF-β–mediated EMT and migration in HeLa cells. Conversely, overexpression of FAD104 suppressed TGF-β–induced EMT. In addition, we showed that FAD104 negatively regulated phosphorylation of Smad2 and Smad3 but positively regulated phosphorylation of Smad1/5/8 via treatment with TGF-β. These findings demonstrate that FAD104 is a novel suppressor of TGF-β signalling and represses TGF-β–mediated EMT in cervical cancer cells.

## Introduction

Metastasis is a typical feature of malignancy. It is well known that metastatic cancer is more difficult to treat than cancer that has not spread^1,2^. Cancer cell metastasis is a multistep process, consisting of local invasion, intravasation, circulation, extravasation and colonization^3,4^. In order to intravasate into blood vessels, metastatic cells undergo epithelial-to-mesenchymal transition (EMT). During EMT, epithelial cells with polarity translate into mesenchymal cells with increased motility and are more likely to move freely in the extracellular matrix, resulting in increased metastatic capabilities^5–7^. EMT is triggered by a variety of soluble factors including epidermal growth factor, hepatocyte growth factor and transforming growth factor-β (TGF-β), and it is regulated by many transcription factors such as Snail, Slug and Twist^8–10^. Recently, research by 2 groups demonstrated that EMT may be more important for the acquisition of chemotherapy resistance than for metastasis in some cancers^11,12^. To identify novel therapeutic targets for cancers, the molecular mechanism involved in the regulation of EMT must be elucidated.

Previously, we isolated 102 genes whose expression was upregulated in the early stages of adipocyte differentiation and we demonstrated that some novel genes including the factor for adipocyte differentiation 24 (fad24), fad49, fad104 and fad158 promoted adipocyte differentiation^13–18^. FAD104 has a proline-rich region, 9 fibronectin type III domains and a transmembrane region and it is also called fibronectin type III domain containing protein (FNDC) 3B^17,19^. Previous analyses using *fad104*-deficient mice showed that *fad104* plays a pivotal role in bone formation and lung maturation in addition to regulating of adipocyte differentiation^20–23^.

We also reported that *fad104* suppressed the invasion and metastasis of melanoma and breast cancer cells by inhibiting the signal transducer and activator of transcription 3 (STAT3) activity^24^. Furthermore, we recently demonstrated that *fad104* suppressed anchorage-independent growth of melanoma cells, and that the N-terminal region of FAD104 was essential for inhibiting malignant transformation and STAT3 activity^25^. These findings strongly suggest that FAD104 is closely associated with cancer cell metastasis. However, it is not known whether FAD104 contributes to the regulation of EMT.

In the present study, we revealed that expression of FAD104 is upregulated during TGF-β–mediated EMT in human cervical cancer HeLa and CaSki cells. Furthermore, a reduction of *fad104* expression enhanced TGF-β–mediated EMT and migration in HeLa cells. In contrary, overexpression of FAD104 suppressed TGF-β–induced EMT. In addition, we showed that FAD104 negatively regulates phosphorylation of Smad2 and Smad3 but positively regulates phosphorylation of Smad1/5/8 via TGF-β treatment. These results indicate that FAD104 is a novel suppressor of TGF-β signalling and represses TGF-β–mediated EMT in cervical cancer cells.

## Results

### Expression of FAD104 is elevated during TGF-β–mediated EMT in HeLa and NMuMG cells

**Figure 1:**
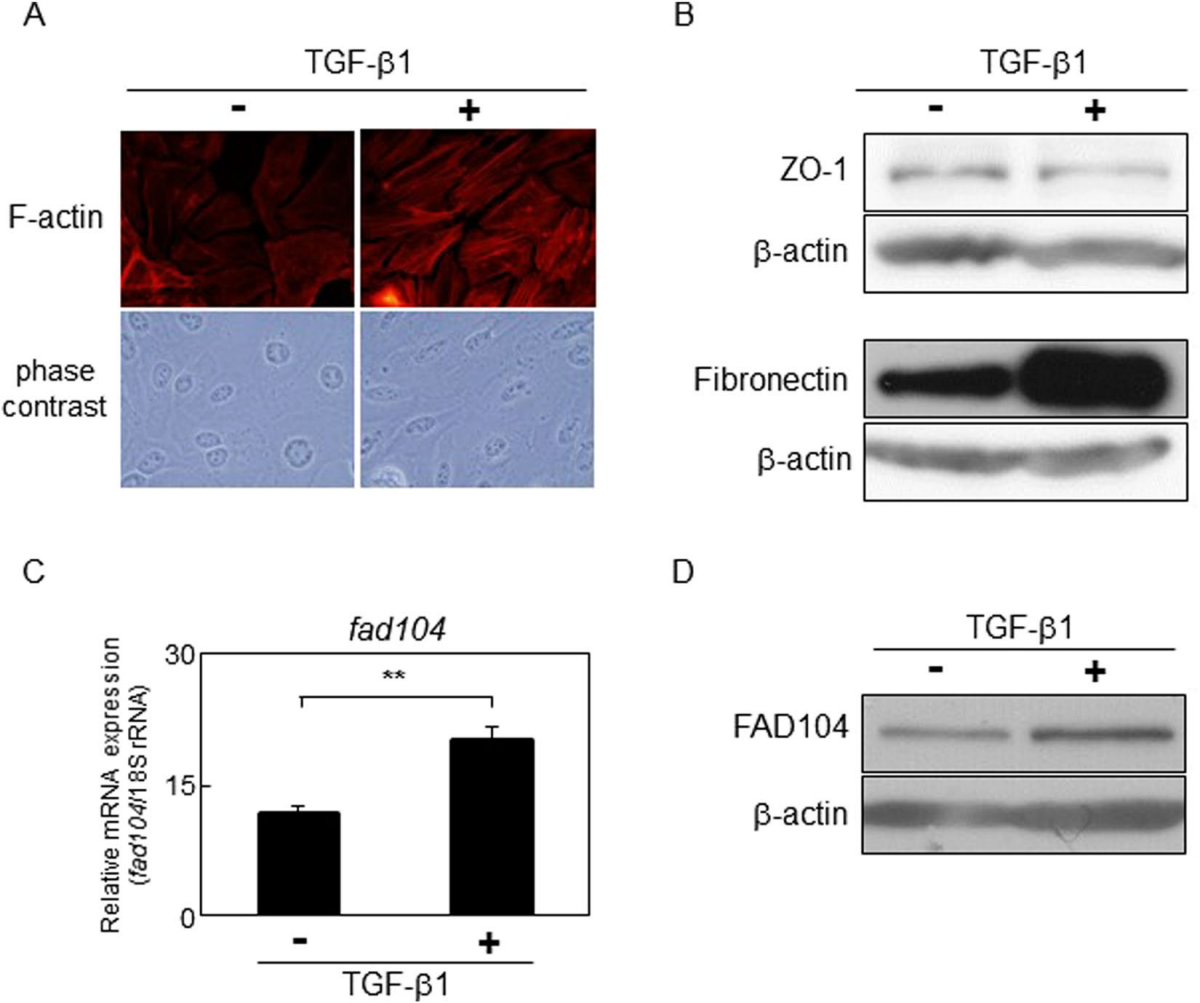
FAD104 expression is elevated during TGF-β–mediated EMT in HeLa cells. HeLa cells were treated with 5 ng/mL TGF-β1 or vehicle for 72 h. (**A**) Morphological changes of HeLa cells after treatment with TGF-β1. F-actin was visualized by TRITC-conjugated phalloidin. (**B**) The expression of the epithelial marker ZO-1 and mesenchymal marker Fibronectin after treatment with TGF-β1. Whole-cell lysates were subjected to Western blot analysis and β-actin was used as a loading control. (**C**) qPCR analysis of *fad104* expression in HeLa cells treated with TGF-β1. The expression level of *fad104* was normalized with 18 S rRNA expression. Each column represents the mean with standard deviation (n = 3). Significant differences are indicated as **p < 0.01. (**D**) Protein expression of FAD104 in HeLa cells after treatment with TGF-β1. Whole-cell lysates were subjected to Western blot analysis and β-actin was used as a loading control.

We first examined the level of expression of FAD104 during TGF-β–mediated EMT in HeLa cells. HeLa cells were treated with TGF-β1 and stained for F-actin with tetramethylrhodamine isothiocyanate (TRITC) –conjugated phalloidin. At 72 hours after treatment with TGF-β1, HeLa cells formed long actin stress fibers and were more elongated than control cells treated with vehicle (Fig. 1A). Furthermore, the expression level of ZO-1, an epithelial marker gene, decreased with TGF-β1 treatment, whereas the expression of fibronectin, a mesenchymal marker, was upregulated (Fig. 1B). These results suggested that TGF-β1 treatment for 72 h induced EMT in HeLa cells. Quantitative real-time polymerase chain reaction (qPCR) and Western blot analyses showed that expression levels of *fad104* in cells treated with TGF-β1 were higher than those in control cells (Fig. 1C and D).

**Figure 2:**
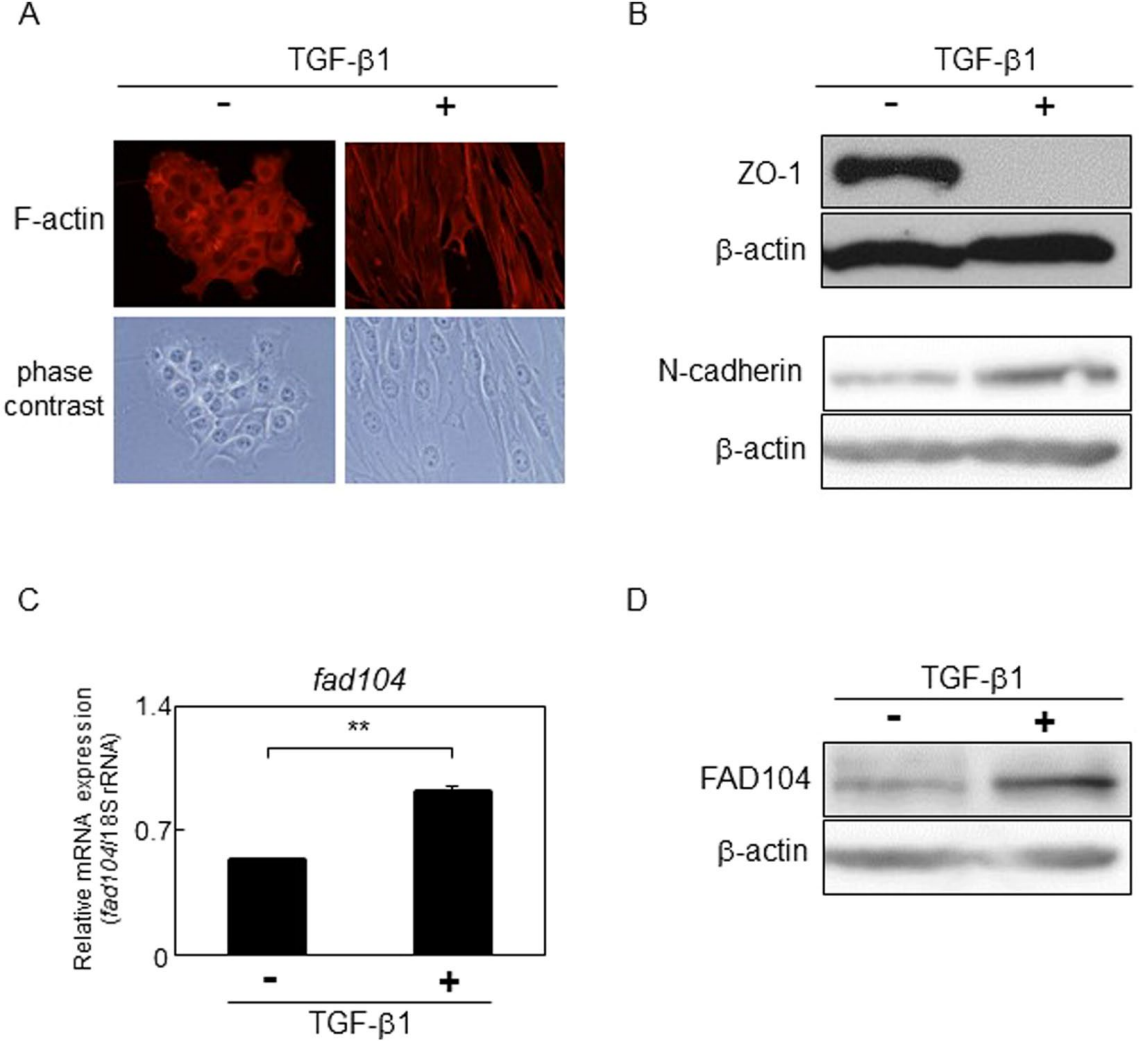
FAD104 expression is elevated during TGF-β–mediated EMT in NMuMG cells. NMuMG cells were treated with 5 ng/mL TGF-β1 or vehicle for 48 h. (**A**) Morphological changes in NMuMG cells after treatment with TGF-β1. F-actin was visualized by TRITC-conjugated phalloidin. (**B**) Expression of the epithelial marker ZO-1 and mesenchymal marker N-cadherin after treatment with TGF-β1. Whole-cell lysates were subjected to Western blot analysis and β-actin was used as a loading control. (**C**) qPCR analysis of *fad104* expression in NMuMG cells treated with TGF-β1. The expression level of *fad104* was normalized with 18 S rRNA expression. Each column represents the mean with standard deviation (n = 3). Significant differences are indicated as **p < 0.01. (**D**) Protein expression of FAD104 in NMuMG cells after treatment with TGF-β1. Whole-cell lysates were subjected to Western blot analysis and β-was used as a loading control.

Similar to HeLa cells, NMuMG cells, a nontransformed mouse mammary gland epithelial cell line, are also frequently used as a model for TGF-β–mediated EMT. We then examined whether expression of FAD104 was upregulated during EMT in NMuMG cells. At 48 hours after treatment with TGF-β1, the number of actin stress fibers notably increased and a morphological change from being cuboidal-shaped to spindle-shaped was observed (Fig. 2A). Moreover, TGF-β1 treatment downregulated the expression of ZO-1 and upregulated the expression of N-cadherin, a mesenchymal marker (Fig. 2B). *Fad104* mRNA and protein expression increased in NMuMG cells treated with TGF-β1 for 48 h (Fig. 2C and D). These results indicate that FAD104 expression is elevated during TGF-β–induced EMT.

### *Fad104* knockdown enhances TGF-β–mediated EMT in cervical cancer cells

**Figure 3:**
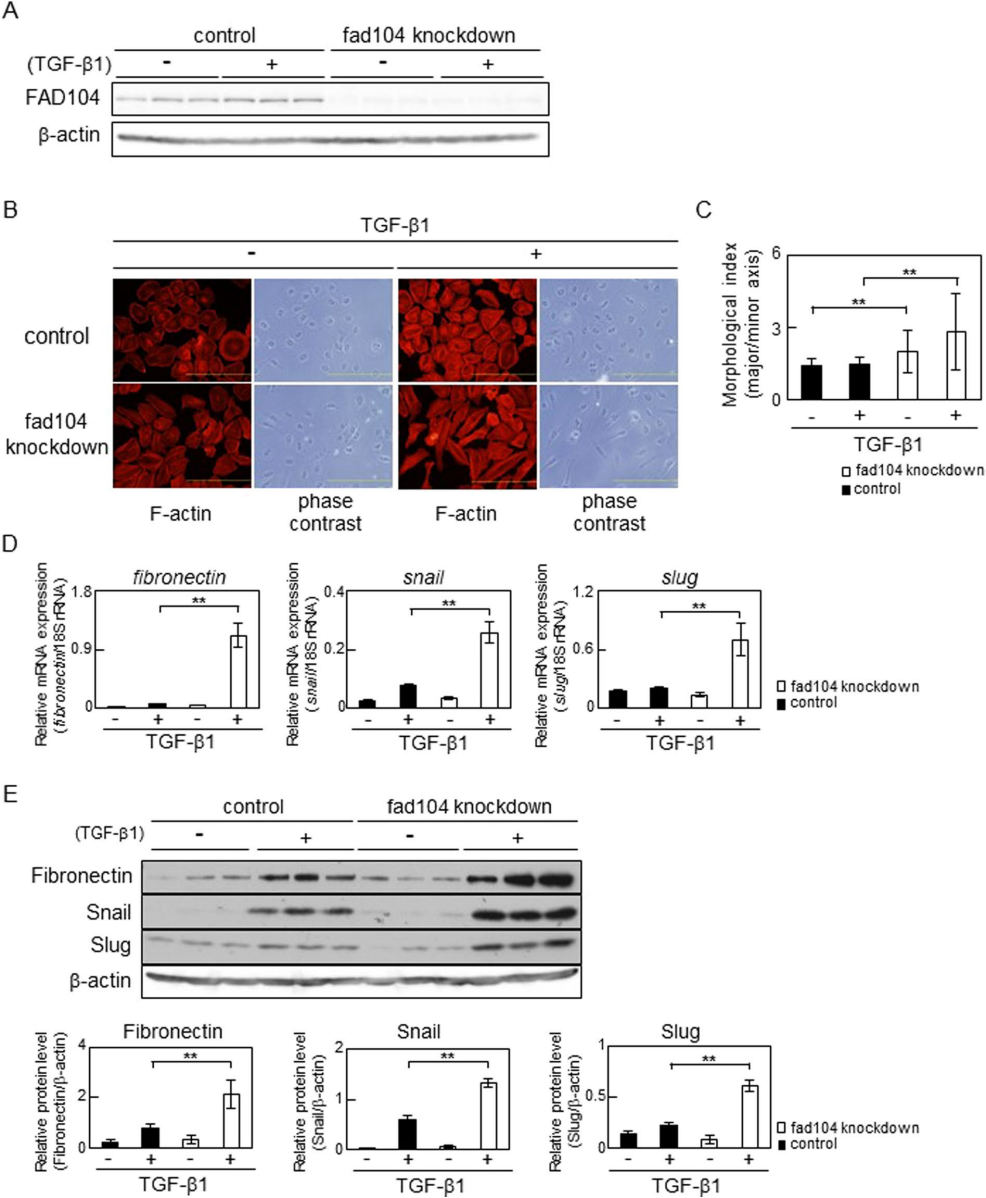
Fad104 knockdown enhances TGF-β–mediated EMT in HeLa cells. (**A**) Knockdown efficiency of *fad104* in HeLa cells. HeLa cells were transfected with siRNA targeting *fad104* (sifad104-A) and treated with 5 ng/mL TGF-β1. Luciferase siRNA was used as a control. β-Actin expression was used as a loading control. (**B**) Morphological changes in HeLa cells transfected with *fad104* siRNA. Cells were treated with 5 ng/mL TGF-β1 for 72 h. F-actin was visualized by TRITC-conjugated phalloidin. Scale bars represent 100 μm. (**C**) Quantitative analysis of cell morphology of HeLa cells in (**B**). The lengths of the major and minor cell axes were measured using NIH-Image software. The ratios of the major to minor axes of cells were used to determine the degree of elongated cell morphology. For each experiment, over 20 cells in each condition were measured. Each column represents the mean with standard deviation. (**D**) qPCR analysis of *fibronectin, snail*, and *slug* expression in *fad104* knockdown cells. Cells were treated with 1 ng/mL TGF-β1 for 72 h. Expression levels of *fibronectin, snail*, and *slug* were normalized with 18 S rRNA expression. Each column represents the mean with standard deviation (n = 3). (**E**) Protein expression of fibronectin, Snail, and Slug in *fad104* knockdown cells. Whole-cell lysates were subjected to Western blot analysis and β-actin was used as a loading control. Signal intensities of the proteins were quantified using NIH-Image software. Each column represents the mean with standard deviation (n = 3). Significant differences are indicated as **p < 0.01.

To clarify the role of *fad104* in EMT, we conducted knockdown experiments. HeLa cells were transfected with a control small interfering RNA (siRNA) or siRNA targeting *fad104* (fad104-A). The protein level of FAD104 decreased by about 90% in *fad104* knockdown cells compared with that in control cells, regardless of the TGF-β1 treatment (Fig. 3A). Next, F-actin of *fad104* knockdown and control cells was stained with TRITC –conjugated phalloidin. After TGF-β1 treatment, the number of long and thick actin stress fibers increased in *fad104* knockdown cells compared with that in control cells (Fig. 3B). Furthermore, the degree of elongated cell morphology significantly increased in *fad104* knockdown cells treated with TGF-β1 compared with that in control cells (Fig. 3C).

We evaluated expression levels of EMT-related genes. TGF-β1 treatment drastically increased *fibronectin* mRNA expression in *fad104* knockdown cells (Fig. 3D). Furthermore, mRNA expression of *snail* and *slug*, transcription factors promoting EMT, was also elevated in *fad104* knockdown cells treated with TGF-β1 (Fig. 3D). In addition, Western blot analyses revealed that protein levels of Fibronectin, Snail and Slug significantly increased in *fad104* knockdown cells treated with TGF-β1 compared with that in control cells (Fig. 3E). Similar results were obtained from knockdown experiments using fad104-B, a siRNA-targeting region different from fad104-A (Supplementary Figure S1). Analyses of cell morphology and the expression of EMT-related genes strongly suggested that reduction of *fad104* expression enhanced TGF-β–induced EMT in HeLa cells.

Next, we tested whether the reduction of *fad104* expression enhanced TGF-β–induced EMT in other cervical cancer cells than HeLa cells. As with HeLa cells, CaSki cells are also used as a model for TGF-β–mediated EMT^26,27^. We first examined the expression level of *fad104* during EMT in CaSki cells. qPCR and Western blot analyses showed that *fad104* expression increased in CaSki cells treated with TGF-β1 for 72 h (Supplementary Figure S2A and S2B). We next examined whether *fad104* regulates TGF-β–induced EMT in CaSki cells. Since the expression of Snail was not detected in CaSki cells treated with or without TGF-β1, we evaluated expression levels of Fibronectin and Slug. As shown in Supplementary Figure S2C, the expression levels of Fibronectin and Slug significantly increased in *fad104* knockdown cells treated with TGF-β1 compared with those in control cells. These results suggested that reduction of *fad104* expression enhanced TGF-β–induced EMT in CaSki cells as well as HeLa cells.

### Overexpression of FAD104 attenuates TGF-β–mediated EMT in HeLa cells

**Figure 4:**
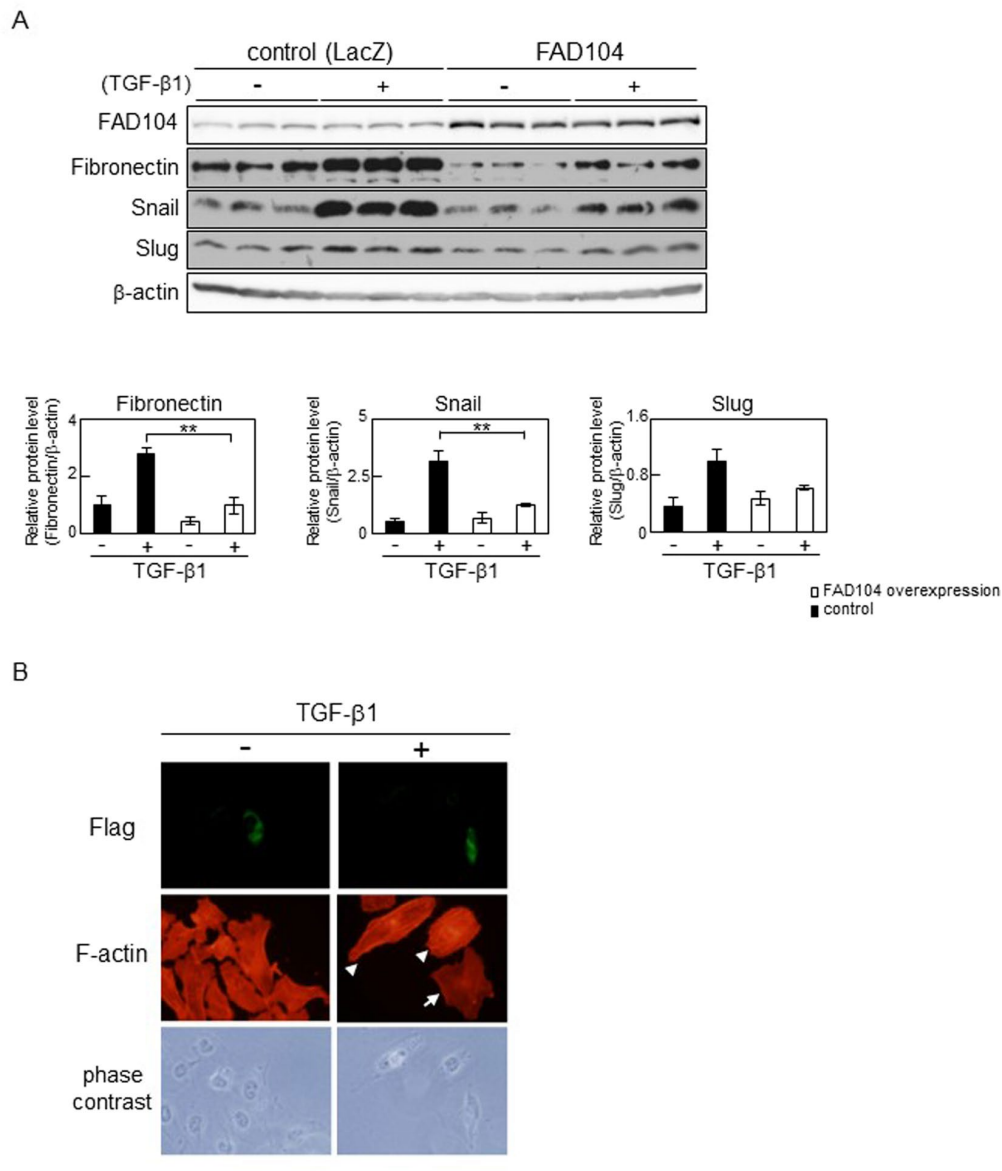
FAD104 overexpression attenuates TGF-β–mediated EMT in HeLa cells. (**A**) The effect of FAD104 overexpression on the expression level of EMT-related genes. HeLa cells were infected with FAD104 or LacZ, and treated with 1 ng/mL TGF-β1 for 72 h. Whole-cell lysates were subjected to Western blot analysis and β-actin was used as a loading control. Signal intensities of the proteins were quantified using NIH-Image software. Each column represents the mean with standard deviation (n = 3). Significant differences are indicated as **p < 0.01. (**B**) Flag-tagged FAD104 expression plasmid was introduced into HeLa cells. After transfection, the cells were treated with 5 ng/mL TGF-β1 for 72 h. The signals of F-actin (red) and Flag-tagged FAD104 (green) were detected with fluorescence microscopy. Arrow indicates the cells expressing Flag-tagged FAD104. Arrowheads indicate untransfected cells as Flag signal is not detected.

We then examined the effect of overexpression of FAD104 on TGF-β–mediated EMT. HeLa cells were infected with adenovirus encoding either FAD104 or LacZ. Exogenous expression of FAD104 in HeLa cells was attained by adenoviral transduction of *fad104* (Fig. 4A). After treatment with TGF-β1, expressions of Fibronectin, Snail and Slug were upregulated in control cells infected with LacZ. FAD104 overexpression significantly suppressed the increased expression of Fibronectin and Snail after the treatment (Fig. 4A). Overexpression of FAD104 also inhibited the increased expression of Slug; however, the difference was not statistically significant (Fig. 4A).

We tested whether FAD104 overexpression influenced stress fiber formation induced by TGF-β1 treatment. HeLa cells were transfected with a Flag-tagged FAD104 expression plasmid. At 72 hours after TGF-β1 treatment, the number of actin stress fibers in the untransfected cells notably increased (Fig. 4B, arrowheads). In contrast, few stress fibers were observed in cells expressing Flag-tagged FAD104 (Fig. 4B, arrow). These results suggested that FAD104 overexpression suppresses TGF-β–mediated EMT in HeLa cells.

### Reduction of *fad104* expression enhances migration of HeLa cells undergoing TGF-β–mediated EMT

**Figure 5:**
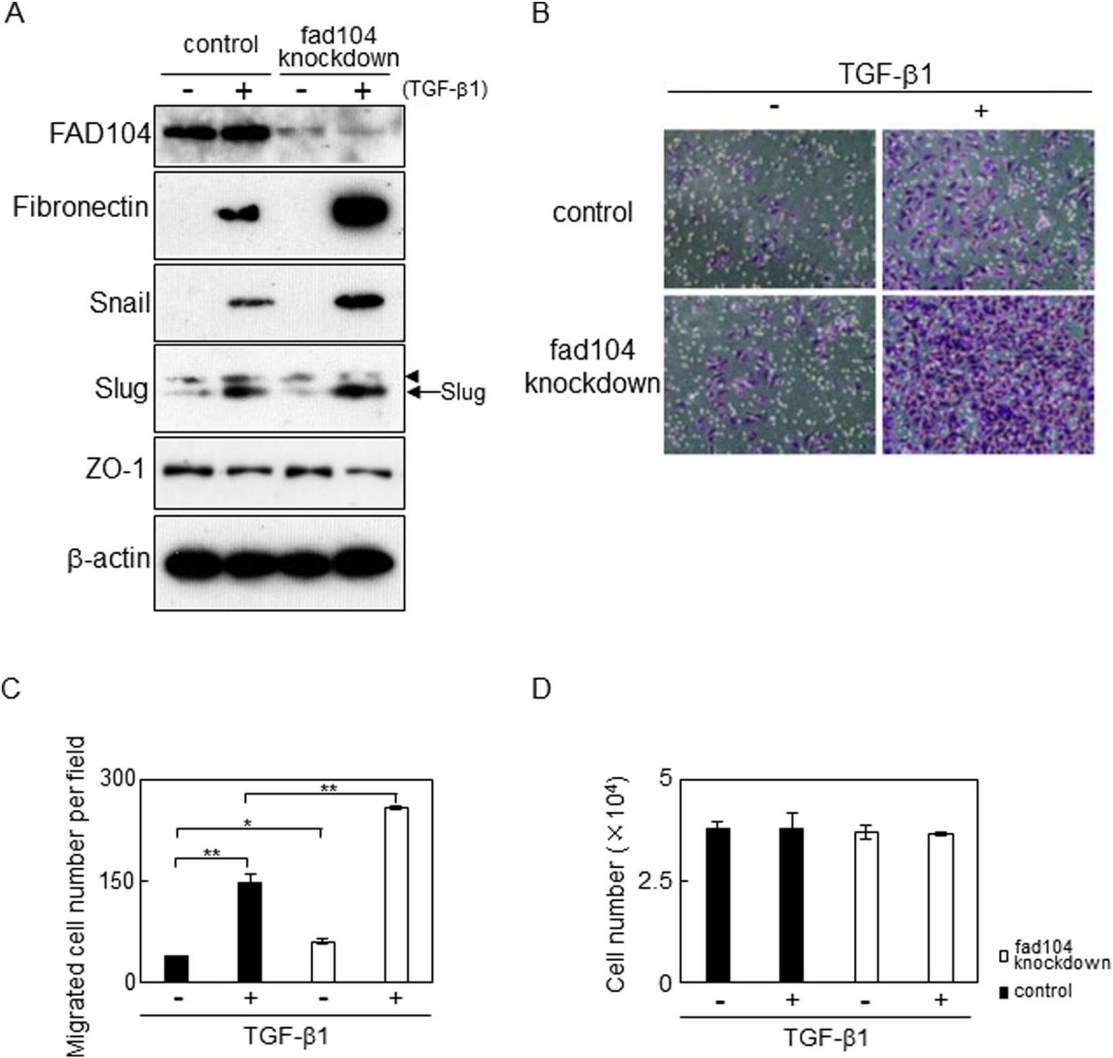
Knockdown of fad104 expression enhances the migration of HeLa cells undergoing TGF-β–mediated EMT. HeLa cells were transfected with siRNA targeting *fad104* (sifad104-A) and treated with 1 ng/mL TGF-β1 for 72 h. (**A**) Whole-cell lysates prepared from HeLa cells undergoing EMT were subjected to Western blot analysis. β-Actin was used as a loading control. Arrowhead shows nonspecific bands. (**B**) Cells undergoing EMT were plated in the upper chamber of the filters coated with fibronectin. Cells that migrated to the underside of the transwell insert were measured after 24 h. Representative images of migrated cells were shown. (**C**) The mean number of migrated cells in the field was calculated. Each column represents the mean with standard deviation (n = 5). (**D**) Cells undergoing EMT were plated in culture plates. After 24 h, cells were trypsinized and counted. Each column represents the mean with standard deviation (n = 3). Significant differences are indicated as **p < 0.01 and *p < 0.05.

It is well known that tumour cells undergoing EMT acquire migratory capacity^8–10^. Therefore, we examined whether *fad104* was involved in the migration of HeLa cells following TGF-β–mediated EMT. HeLa cells were transfected with control siRNA or fad104 siRNA (sifad104) and treated with TGF-β1 for 72 h to induce EMT. We first examined the expression levels of EMT markers. The expression of fibronectin and Snail increased in *fad104* knockdown cells treated with TGF-β1 compared with that in control cells. Slug expression slightly increased in *fad104* knockdown cells treated with TGF-β1. On the other hand, the expression of these proteins was not elevated in *fad104* knockdown cells treated with vehicle (Fig. 5A). In addition, an epithelial marker ZO-1 expression was reduced in *fad104* knockdown cells treated with TGF-β1, suggesting that these cells were underwent EMT by treatment with TGF-β1 (Fig. 5A). Next, migration assay using fibronectin-coated transwell chambers was performed. The reduction of *fad104* expression increased the migration ability of HeLa cells that had undergone TGF-β–mediated EMT. Furthermore, *fad104* knockdown also facilitated migration of HeLa cells treated with vehicle (Fig. 5B and C). To examine whether the change of cell proliferation rate influences on the number of migrated cells, we performed cell proliferation assay. As a result, *fad104* knockdown had little influence on the growth of HeLa cells undergoing EMT (Fig. 5D). These results suggest that FAD104 contributes to regulating migration of HeLa cells that have undergone EMT.

### FAD104 regulates TGF-β–Smad signalling

**Figure 6:**
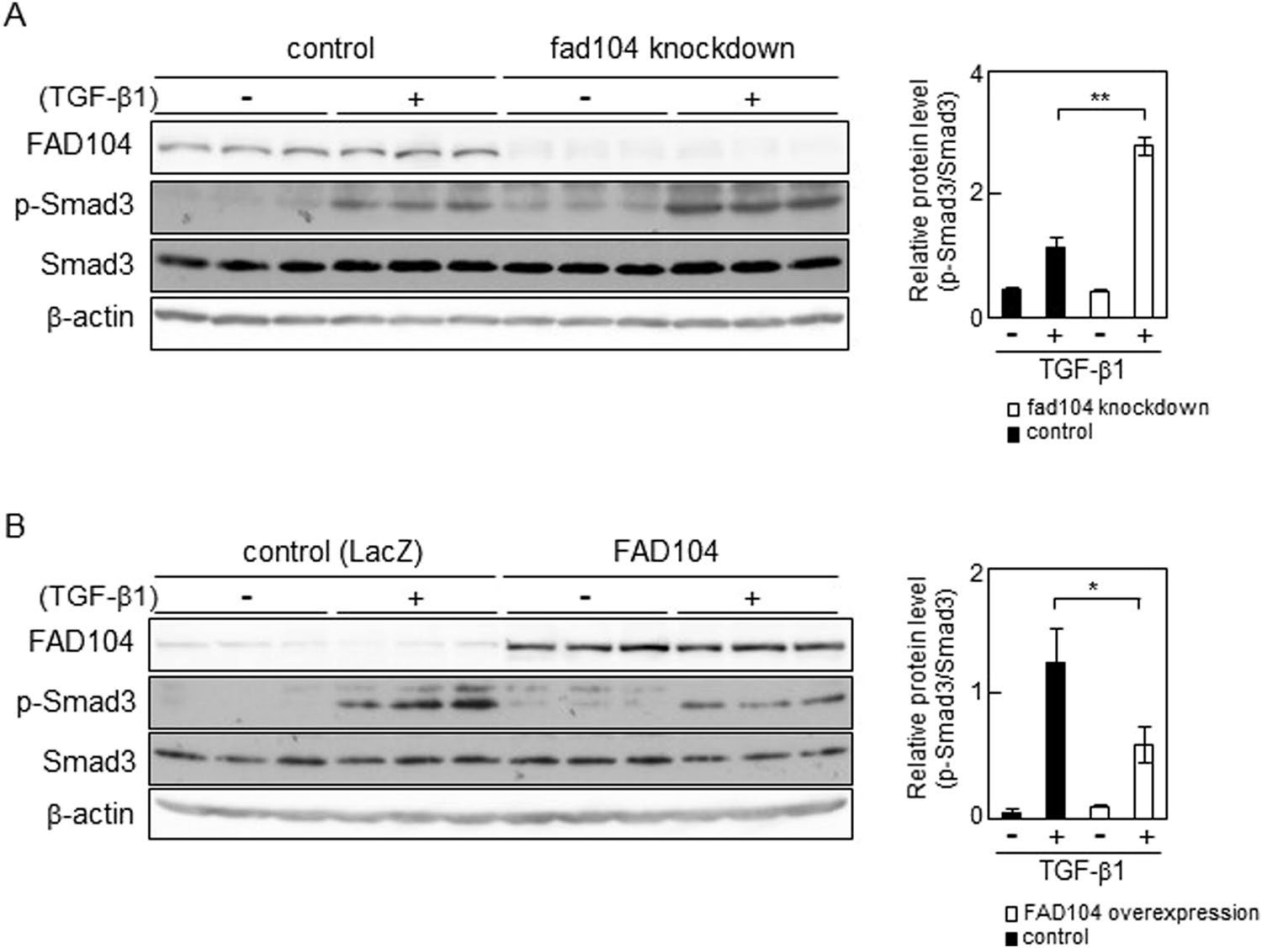
FAD104 negatively regulates phosphorylation level of Smad3 with TGF-β1 treatment in HeLa cells. (**A**) Phosphorylation levels of Smad3 in *fad104* knockdown HeLa cells. HeLa cells were transfected with siRNA targeting *fad104* (sifad104-A) and treated with 1 ng/mL TGF-β1 for 6 h. (**B**) Phosphorylation levels of Smad3 in HeLa cells overexpressing FAD104. HeLa cells were infected with FAD104 and treated with 1 ng/mL TGF-β1 for 6 h. Whole-cell lysates were subjected to Western blot analysis and β-actin was used as a loading control. Signal intensities from phospho-Smad3, total Smad3, and β-actin were quantified using NIH-Image software. Each column represents the mean with standard deviation (n = 3). Significant differences are indicated as **p < 0.01 and *p < 0.05.

TGF-β binds to its receptors and leads to phosphorylation of Smad2 and Smad3^28,29^. We examined whether FAD104 contributed to the regulation of Smad3 phosphorylation during TGF-β–mediated EMT. The level of phosphorylation in Smad3 significantly increased in *fad104* knockdown cells treated with TGF-β1 compared with that in control cells, while the level of total Smad3 did not differ between *fad104* knockdown cells and control cells (Fig. 6A). Furthermore, FAD104 overexpression significantly decreased Smad3 phosphorylation after treatment with TGF-β1 (Fig. 6B). We next tested whether the reduction of *fad104* expression influenced on the phosphorylation level of Smad2 during TGF-β-mediated EMT. The level of phosphorylation in Smad2 significantly increased in *fad104* knockdown cells treated with TGF-β1 compared with that in control cells (Supplementary Figure S3).

**Figure 7:**
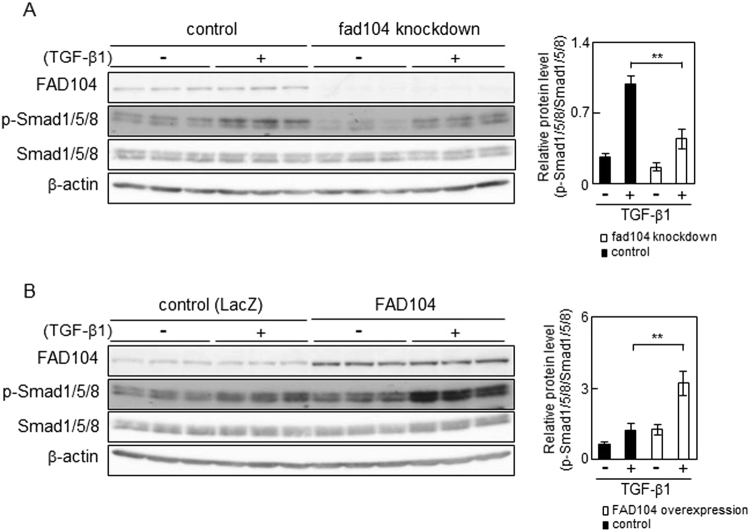
FAD104 positively regulates phosphorylation level of Smad1/5/8 with TGF-β1 treatment in HeLa cells. (**A**) Phosphorylation levels of Smad1/5/8 in *fad104* knockdown HeLa cells. HeLa cells were transfected with siRNA targeting *fad104* (sifad104-A) and treated with 1 ng/mL TGF-β1 for 30 min. (**B**) Phosphorylation levels of Smad1/5/8 in HeLa cells overexpressing FAD104. HeLa cells were infected with FAD104 and treated with 1 ng/mL TGF-β1 for 30 min. Whole-cell lysates were subjected to Western blot analysis and β-actin was used as a loading control. Signal intensities from phospho-Smad1/5/8, total Smad1/5/8, and β-actin were quantified using NIH-Image software. Each column represents the mean with standard deviation (n = 3). Significant differences are indicated as **p < 0.01.

It is reported that TGF-β can additionally activate Smad1 and Smad5 in various cells^30–32^. Therefore, we tested the level of phosphorylation in Smad1/5/8 during TGF-β–induced EMT. Knockdown and overexpression experiments showed that FAD104 facilitates Smad1/5/8 phosphorylation induced by TGF-β1 (Fig. 7). These results suggested that *fad104* negatively regulated Smad2 and Smad3 phosphorylation but positively regulated Smad1/5/8 phosphorylation during TGF-β–induced EMT in HeLa cells.

## Discussion

Despite improvements in diagnosis and screening techniques, cervical cancer is one of the most common malignant tumours and remains the second-largest cause of cancer-related deaths in women worldwide^33^. Especially, pelvic lymph node metastasis is an important risk factor for recurrence or death in cervical cancer^34,35^. Although it is thought that EMT plays a role in acquisition of the ability to invade the pelvic lymph nodes and surrounding tissue, the regulatory mechanisms of EMT in cervical cancer are not fully understood. In this study, we demonstrated for the first time that *fad104* functions as a novel suppressor of TGF-β–mediated EMT in cervical cancer cells.

Knockdown and overexpression experiments showed that FAD104 negatively regulates phosphorylation of Smad2 and Smad3 after treatment with TGF-β (Fig. 6). Furthermore, expression levels of FAD104 were elevated in HeLa and NMuMG cells treated with TGF-β (Figs 1 and 2). It is well known that TGF-β signalling is precisely regulated by a balance between stimulatory factors such as Smad2 and Smad3 and inhibitory factors including SnoN and Smad7. The expression of Smad7 and SnoN is upregulated by TGF-β and they act as negative feedback regulators of TGF-β signalling^36^. Yin *et al*. demonstrated that SnoN suppressed TGF-β–induced EMT and invasion of bladder cancer^37^. Moreover, it was reported that some microRNAs targeting Smad7 promote TGF-β–mediated EMT and invasion of cancer cells^38,39^. These findings raise the possibility that FAD104 also participates in a negative feedback loop to regulate TGF-β signalling and limits the extended activation of TGF-β signalling leading to EMT.

Our previous report showed that FAD104 expression was lower in highly metastatic melanoma cells than in poorly metastatic cells^24^. Furthermore, in the Human Protein Atlas (www.proteinatlas.org), expression levels of FAD104 in most malignant tumours including cervical and breast cancer are lower than those in corresponding normal tissues. Therefore, it is possible that the reduction in FAD104 expression is deeply involved in the malignancy of cancer cells such as cervical cancer and melanoma. Promoter analysis is required to determine how TGF-β upregulates the expression of *fad104*. In addition, we need to perform single-nucleotide polymorphism analyses of the *fad104* promoter in various cancer cells.

As shown in Figs 6 and 7, FAD104 upregulated TGF-β–induced phosphorylation of Smad1/5/8, but it negatively regulated Smad2 and Smad3 phosphorylation. TGF-β signals via the TGF-β type I receptor/activing receptor-like kinase (ALK5) and activates Smad2/3. TGF-β also signals through another type I receptor, ALK1, and activates Smad1/5/8^9,36^. However, the role of Smad1/5/8 activated by TGF-β remains controversial. Goumans *et al*. demonstrated that ALK1 activity not only induces a biological response opposite to that of ALK5 but also directly antagonizes ALK5/Smad signalling in endothelial cells^31^. On the other hand, Daly *et al*. showed that Smad2/3 signalling is not inhibited by Smad1/5 activation^30^. It is necessary to clarify the role and function of Smad1/5/8 activated by TGF-β during EMT of cervical cancer cells.

We previously demonstrated that FAD104 interacted with Smad1/5/8 and down-regulated BMP2-induced Smad1/5/8 phosphorylation in C2C12 cells^20^. Furthermore, our present results suggested that FAD104 negatively regulated Smad2 and Smad3 phosphorylation but positively regulated Smad1/5/8 phosphorylation during TGF-β–induced EMT in HeLa cells (Figs 6 and 7). These findings raise the possibility that FAD104 regulates two Smad signalling pathway though the interaction with Smad proteins. Therefore, we conducted immunoprecipitation experiments. IP using anti-Smad1/5/8 or anti-Smad3 antibody showed that FAD104 does not interact with either Smad1/5/8 or Smad3 in HeLa cells treated with or without TGF-β1 (Supplementary Figure S4). Considering this result, at least in HeLa cells, it seems that FAD104 regulates the phosphorylation levels of Smad1/5/8 and Smad3 other than through the interaction with these proteins. To clarify the role of FAD104 on TGF-β-mediated EMT, we conducted experiments using LY2157299, a TGF-β type I receptor kinase inhibitor. Previous report showed that 5 μM LY2157299 inhibited TGF-β-Smad2/3 signalling in breast cancer cell lines^40^. We first examined the effect of LY2157299 on the phosphorylation level of Smad3. As shown in Supplementary Figure S5A, 5 μM LY2157299 inhibited Smad3 phosphorylation in HeLa cells treated with TGF-β1. We next examined the effect of LY2157299 on the expression levels of mesenchymal markers in control and *fad104* knockdown cells. LY2157299 fully inhibited Fibronectin and Snail expression elevated by TGF-β1 in both of control and *fad104* knockdown cells, suggesting that FAD104 regulates TGF-β-mediated EMT at least through the control of TGF-β-Smad2/3 signalling (Supplementary Figure S5B). Our previous findings showed that FAD104 down-regulated Smad1/5/8 phosphorylation in C2C12 cells^20^. On the other hand, FAD104 inhibited the phosphorylation level of STAT3, whereas FAD104 had no influence on the Smad1/5/8 phosphorylation in A375 melanoma cells^24^. Therefore, it is possible that FAD104 has different roles and functions in each cell. To more clearly understand the roles of Smad2/3 and Smad1/5/8 in the regulation of FAD104 during TGF-β-mediated EMT, experiments of overexpression and knockdown of Smad2/3 and Smad1/5/8 are needed.

Recently, Vorstenbosch *et al*. reported that CD109, a glycosylphosphatidylinositol (GPI)-anchored protein, directly interacts with ALK5 and ALK1 and inhibits Smad2/3 activation while enhancing Smad1/5 activation in keratinocytes^41^. On the other hand, it was reported that reduced levels of expression of Bmal1, an essential clock transcription activator, increased TGF-β–mediated phosphorylation levels of Smad1/5/8 but decreased phosphorylation levels of Smad2/3 in primary human articular chondrocytes and C3H10T1/2 cells^42,43^. Moreover, loss of neuropilin-1, a coreceptor of semaphorin and vascular endothelial growth factor, elevated phosphorylation levels of Smad1/5/8 but suppressed Smad2/3 phosphorylation in stromal fibroblasts^44^. Although it is unclear how CD109, BMAL1 and neuropilin-1 control phosphorylation levels of Smad1/5/8 and Smad3 with TGF-β treatment, these reports strongly suggest the importance of controlling the balance between TGF-β–mediated ALK5-Smad2/3 and ALK1-Smad1/5/8 signalling. To analyse the relationship between these proteins and FAD104 in TGF-β–induced EMT of cervical cancer cells, we clarify the molecular mechanism by which FAD104 regulates the phosphorylation of Smad1/5/8 and Smad3 in the next paper.

As shown in Fig. 5, the reduction of *fad104* expression increased the migration ability of HeLa cells that had undergone TGF-β–mediated EMT. Furthermore, *fad104* knockdown also facilitated migration of HeLa cells treated with vehicle. Since the expression levels of EMT related markers in *fad104* knockdown cells treated with vehicle did not differ from those in control cells (Fig. 5A), it seems that FAD104 also regulates the migration ability of cells that had not undergone EMT. Our previous report showed that FAD104 suppressed the TGF-β–independent migration of melanoma cells by inhibiting activation of the STAT3 signaling pathway^24^. Liu *et al*. demonstrated that JAK/STAT3 signalling is required for TGF-β-mediated EMT in lung cancer cells^45^. Therefore, we next examined whether FAD104 regulated STAT3 phosphorylation during TGF-β–mediated EMT in HeLa cells. As a result, the knockdown of *fad104* did not influence on the phosphorylation level of STAT3 in HeLa cells treated with both vehicle and TGF-β1 (Supplementary Figure S6). This result suggests that unlike in melanoma cells, FAD104 does not contribute to the regulation of STAT3 activity in HeLa cells. The role of FAD104 in the regulation of migration ability in various cancer cells needs to be investigated.

Considering results obtained from knockdown and overexpression experiments, it seemed that FAD104 has greater effect on the expression of Snail than that of Slug. It is needed to clarify the molecular mechanism by which FAD104 regulates the expression levels of Snail and Slug in TGF-β-mediated EMT in future studies.

In summary, we revealed that expression of FAD104 is upregulated during TGF-β–mediated EMT in HeLa, CaSki and NMuMG cells. Furthermore, the reduction of *fad104* expression enhanced TGF-β–mediated EMT and migration in HeLa cells. Overexpression experiments showed that FAD104 suppressed TGF-β–induced EMT in HeLa cells. In addition, we demonstrated that FAD104 negatively regulated TGF-β–induced phosphorylation of Smad2 and Smad3 but positively regulated phosphorylation of Smad1/5/8. These results strongly suggest that FAD104 suppresses TGF-β–mediated EMT in cervical cancer cells through the control of TGF-β–Smad signalling.

## Materials and Methods

### Cell culture

HeLa cells were purchased from RIKEN Cell Bank and cultured in Minimum Essential Medium (MEM) containing 10% calf serum. NMuMG cells were kindly provided by Dr. Yasumichi Inoue (Nagoya City University) and maintained in high-glucose Dulbecco’s modified Eagle’s medium supplemented with 10% fetal bovine serum (FBS) and 10 μg/mL insulin. CaSki cells were purchased from JCRB Cell Bank and cultured in RPMI1640 containing 10% FBS. Recombinant human TGF-β1 was purchased from R&D systems.

### RNA interference experiments

Two different human fad104 siRNAs (sifad104-A and sifad104-B) and control siRNA were purchased from Nippon EGT and introduced into HeLa cells using Lipofectamine2000 as previously described^24^.

### Adenoviral infection

Adenoviral infection has been described previously^24^. HeLa cells were infected with recombinant adenoviruses expressing FAD104 or LacZ by incubation with adenoviruses at a multiplicity of infection of 200.

### F-actin staining and quantification of elongated cell morphology

HeLa cells transfected with control siRNA or sifad104 were treated with TGF-β1 for 72 h. After washing, cells were fixed for 10 min in 2% paraformaldehyde and stained with TRITC-conjugated phalloidin for detection of the F-actin structure. Measurements of elongated cell morphology were performed as previously reported with slight modifications^46^. The lengths of the major and minor cell axes were measured using NIH-Image software. The ratios of the major to minor axes of cells were used to determine the degree of elongated cell morphology. For each experiment, over 20 cells in each condition were measured.

### Quantitative real time PCR

Total RNA was extracted with RNAiso Plus (TaKaRa) according to the manufacturer’s instructions. Reverse transcription and qPCR were performed as described^47^. The specific primers for human *snail*, *slug*, and *fibronectin* were as follows: human *snail*-forward, 5′-cctcaagatgcacatccgaag-3′; human *snail*-reverse, 5′-acatggccttgtagcagcca-3′; human *slug*-forward, 5′-cccacacattaccttgtgtttgcaa-3′; human *slug*-reverse, 5′-caaatgctctgttgcagtgagg-3′; human *fibronectin*-forward, 5′-gtgttgggaatggtcgtggggaatg-3′; human *fibronectin*-reverse, 5′-ccaatgccacggccatagcagtagc-3′. The predesigned primers and probe sets for *fad104* and 18 S rRNA were obtained from Applied Biosystems.

### Western blotting

Cells were lysed in radioimmunoprecipitation assay buffer as previously described^47^. Equal amounts of total protein were resolved using sodium dodecyl sulfate-polyacrylamide gel electrophoresis and were transferred to a polyvinylidene difluoride membrane, and probed using primary antibodies and secondary antibodies conjugated with horseradish peroxidase (Jackson ImmunoResearch Laboratories). Specific proteins were detected using an enhanced chemiluminescence system (GE Healthcare). Primary antibodies recognizing Fibronectin, ZO-1, N-cadherin, Snail, Slug, phospho-Smad3, Smad3, phospho-Smad1/5/8, Smad1/5/8, phospho-Smad2, phospho-STAT3, STAT3 (Cell Signaling Technology), and β-actin (SIGMA) were used. Primary antibody recognizing Smad2 was kindly provided by Dr. Yasumichi Inoue (Nagoya City University). A polyclonal FAD104 antibody was prepared in our laboratory^21^. Quantification of the band intensity of the blots was performed using NIH-Image software.

### Migration assays

Migration assays using transwell plates were performed as previously reported with slight modifications^48^. After the addition of TGF-β1 for 72 h, half of the cells were lysed and used for Western blotting to analyze the expression levels of EMT related markers. The other half of cells were used for migration and cell proliferation assays. For migration assay, HeLa cells transfected with control siRNA or sifad104 were trypsinized and added to fibronectin-coated inserts in serum-free medium. These were then placed in MEM with 10% calf serum for 24 h. Cells on the upper surface of the membrane were removed by scrubbing with cotton swabs. Chambers were fixed in 4% paraformaldehyde for 10 min and stained with crystal violet. Cells that penetrated the filter were observed with a microscope and cells from 5 randomly fields were counted. For cell proliferation assay, the cells were seeded into 24-well tissue culture plates. After 24 h, numbers of cells were counted.

### Statistical tests

Analyses were performed using Excel 2010 and R (http://cran.r-project.org/). The statistical significance of differences between 2 groups was evaluated using a two-tailed Student’s *t*-test. For the multigroup analysis, significance was assessed using one-way ANOVA with *post hoc* Tukey–Kramer HSD test.

## Electronic supplementary material

Supplementary Information
