# Supplementary material for: FAD104, a regulator of adipogenesis, is a novel suppressor of TGF-β–mediated EMT in cervical cancer cells
Source: Sci Rep. 2017 Nov 27;7:16365. doi: 10.1038/s41598-017-16555-3 (PMC5703855; doi:10.1038/s41598-017-16555-3)

**FAD104, a regulator of adipogenesis, is a novel suppressor of TGF-β–mediated EMT in cervical cancer cells**

Motoharu Goto, Shigehiro Osada, Masayoshi Imagawa and Makoto Nishizuka*

Department of Molecular Biology, Graduate School of Pharmaceutical Sciences, Nagoya City University, 3-1 Tanabe-dori, Mizuho-ku, Nagoya, Aichi 467-8603, Japan

**Supplementary Figure S1. Fad104 knockdown using sifad104-B enhances TGF-β–mediated EMT in HeLa cells.**

(A) Knockdown efficiency of *fad104* in HeLa cells. HeLa cells were transfected with siRNA targeting *fad104* (sifad104-B) and treated with 5 ng/mL TGF-β1. Luciferase siRNA was used as a control. β-Actin expression was used as a loading control. (B) Morphological changes in HeLa cells transfected with *fad104* siRNA. Cells were treated with 5 ng/mL TGF-β1 for 72 h. F-actin was visualized by TRITC-conjugated phalloidin. Scale bars represent 100 μm. (C) Quantitative analysis of cell morphology of HeLa cells in (B). The lengths of the major and minor cell axes were measured using NIH-Image software. The ratios of the major to minor axes of cells were used to determine the degree of elongated cell morphology. For each experiment, over 20 cells in each condition were measured. Each column represents the mean with standard deviation. (D) qPCR analysis of *fibronectin, snail*, and *slug* expression in *fad104* knockdown cells. Cells were treated with 1 ng/mL TGF-β1 for 72 h. Expression levels of *fibronectin, snail*, and *slug* were normalized with 18S rRNA expression. Each column represents the mean with standard deviation (n = 3). (E) Protein expression of fibronectin, Snail, and Slug in *fad104* knockdown cells. Whole-cell lysates were subjected to Western blot analysis and β-actin was used as a loading control. Signal intensities of the proteins were quantified using NIH-Image software. Each column represents the mean with standard deviation (n = 3). Significant differences are indicated as ***p* < 0.01 and **p* < 0.05.


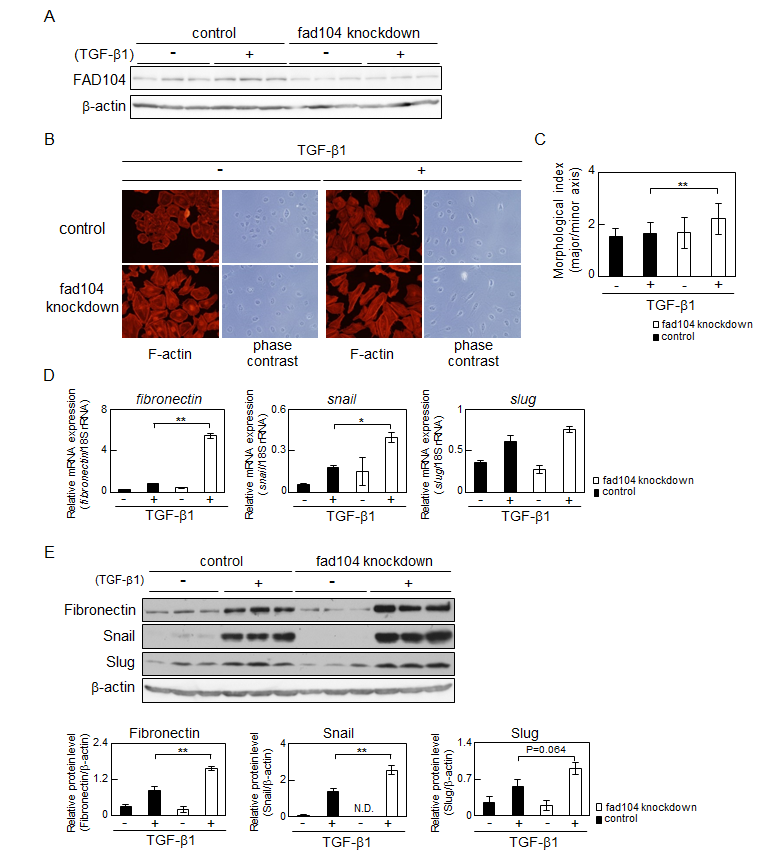


**Supplementary Figure S2. *Fad104* knockdown enhances TGF-β–mediated EMT in CaSki cells.**

CaSki cells were treated with 1 ng/mL TGF-β1 or vehicle for 72 h. (A) qPCR analysis of *fad104* expression in CaSki cells treated with TGF-β1. The expression level of *fad104* was normalized with 18S rRNA expression. Each column represents the mean with standard deviation (n = 3). Significant differences are indicated as ***p* < 0.01. (B) Protein expression of FAD104 in CaSki cells after treatment with TGF-β1. Whole-cell lysates were subjected to Western blot analysis and β-actin was used as a loading control. Signal intensities of the proteins were quantified using NIH-Image software. Each column represents the mean with standard deviation (n = 3). Significant differences are indicated as ***p* < 0.01. (C) Protein expression of fibronectin and Slug in *fad104* knockdown cells. Whole-cell lysates were subjected to Western blot analysis and β-actin was used as a loading control. Signal intensities of the proteins were quantified using NIH-Image software. Each column represents the mean with standard deviation (n = 3). Significant differences are indicated as ***p* < 0.01.


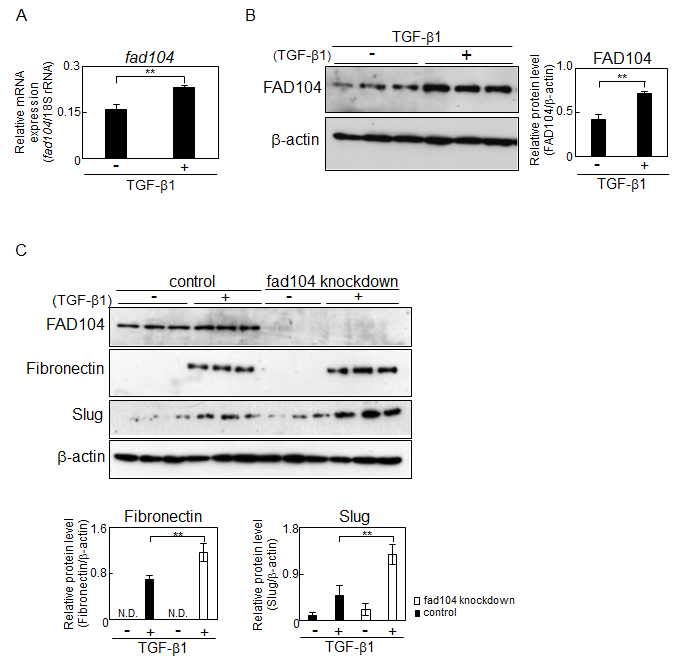


**Supplementary Figure S3. Knockdown of fad104 expression enhances phosphorylation level of Smad2 with TGF-β1 treatment in HeLa cells.** Phosphorylation levels of Smad2 in *fad104* knockdown HeLa cells. HeLa cells were transfected with siRNA targeting *fad104* (sifad104-A) and treated with vehicle or TGF-β1 for 6 h. Signal intensities from phospho-Smad2, total Smad2, and β-actin were quantified using NIH-Image software. Each column represents the mean with standard deviation (n = 3). Significant differences are indicated as ***p* < 0.01.


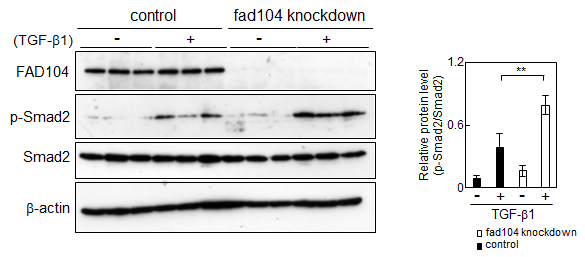


**Supplementary Figure S4. FAD104 does not interact with either Smad1/5/8 or Smad3 in HeLa cells treated with or without TGF-β1. (**A and B) Immunoprecipitation (IP) assay using anti-Smad1/5/8 antibody. HeLa cells were treated with 1 ng/mL TGF-β1 (A) or vehicle (B) for 30 min and lysed with Nonidet-P40 lysis buffer. IP experiments were performed using anti-Smad1/5/8 antibody. Normal rabbit IgG was used as a negative control. Immunoprecipitates and inputs were resolved and detected by Western blotting with anti-FAD104 antibody. (C and D) IP assay using anti-Smad3 antibody. HeLa cells were treated with 1 ng/mL TGF-β1 (C) or vehicle (D) for 6 h and lysed with Nonidet-P40 lysis buffer. IP experiments were performed using anti-Smad3 antibody. Normal rabbit IgG was used as a negative control. Immunoprecipitates and inputs were resolved and detected by Western blotting with anti-FAD104 antibody.


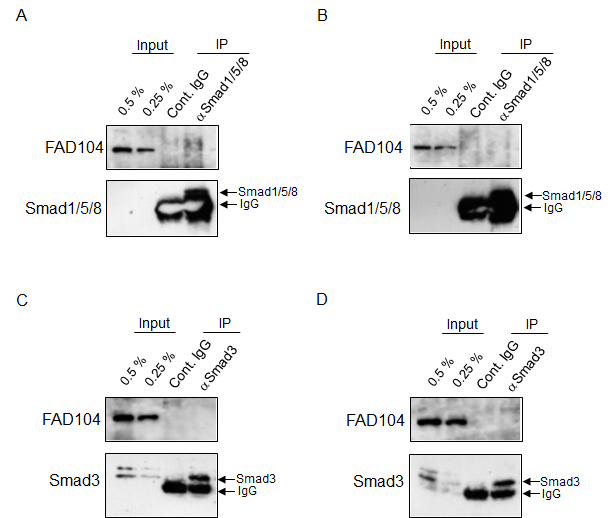


**Supplementary Figure S5. LY2157299, a TGF-β type I receptor kinase inhibitor, inhibits Fibronectin and Snail expression elevated by TGF-β1 in both of control and *fad104* knockdown cells.** (A) Phosphorylation levels of Smad3 in HeLa cells treated with LY2157299. HeLa cells were pre-treated with or without LY2157299 for 30 min followed by stimulation with vehicle or TGF-β1 for 6 h. (B) Protein expression of fibronectin and Snail in *fad104* knockdown cells treated with LY2157299. HeLa cells were transfected with siRNA targeting *fad104* (sifad104-A) and pre-treated with or without LY2157299 for 30 min followed by stimulation with vehicle or TGF-β1 for 72 h. Whole-cell lysates were subjected to Western blot analysis and β-actin was used as a loading control.


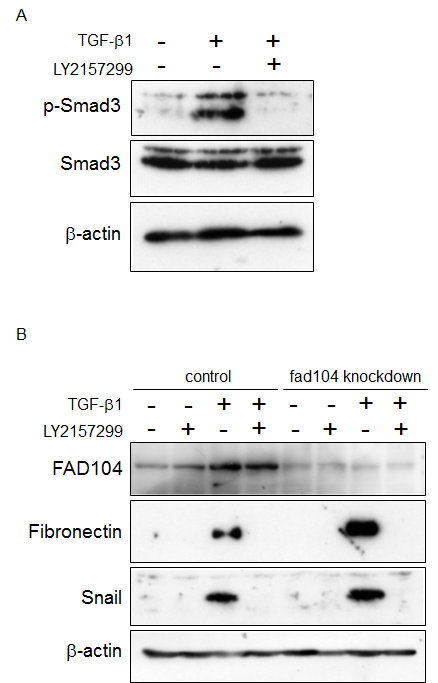


**Supplementary Figure S6. FAD104 does not contribute to the regulation of STAT3 phosphorylation level in HeLa cells.** Phosphorylation levels of STAT3 in *fad104* knockdown HeLa cells. HeLa cells were transfected with siRNA targeting *fad104* (sifad104-A) and treated with vehicle or TGF-β1 for 6 h. Signal intensities from phospho-STAT3, total STAT3, and β-actin were quantified using NIH-Image software. Each column represents the mean with standard deviation (n = 3).


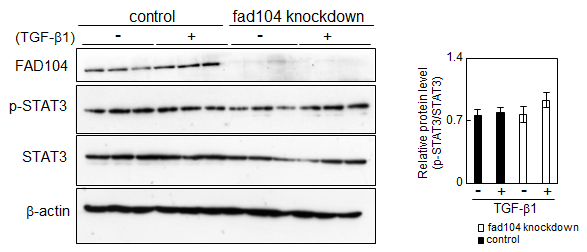


**Supplementary Figure S7. Original uncropped images of blots.**

Boxes highlighted lanes used in Fig. 1.


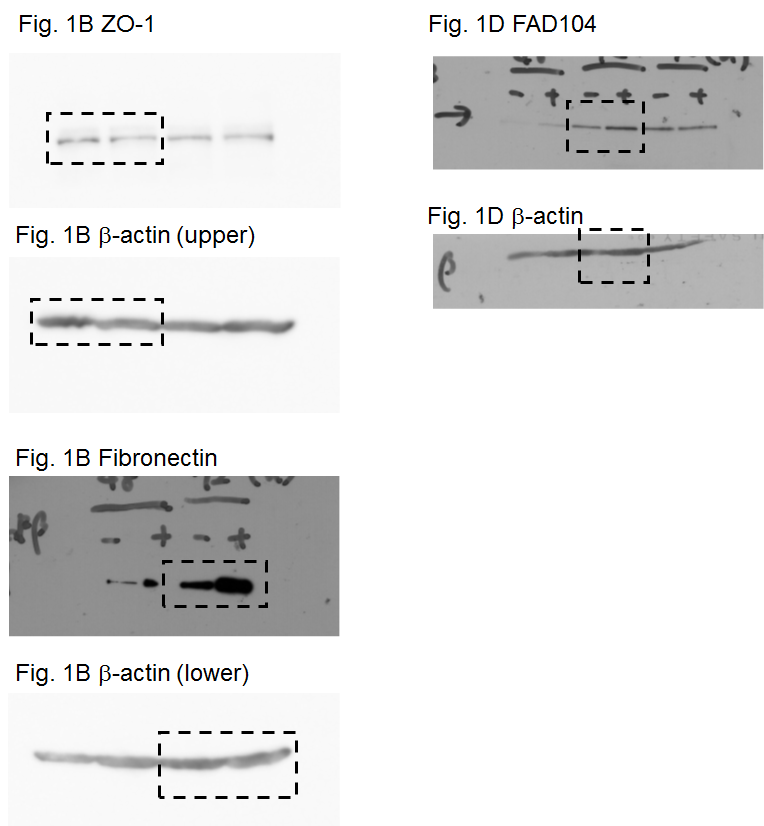


**Supplementary Figure S8. Original uncropped images of blots.**

Boxes highlighted lanes used in Fig. 2.


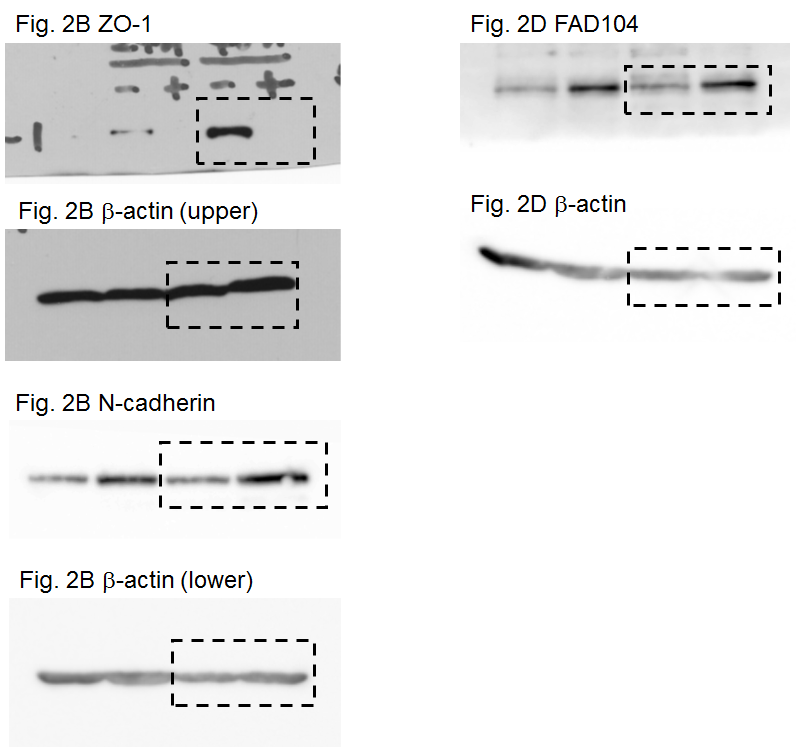


**Supplementary Figure S9. Original uncropped images of blots.**

Boxes highlighted lanes used in Fig. 3.


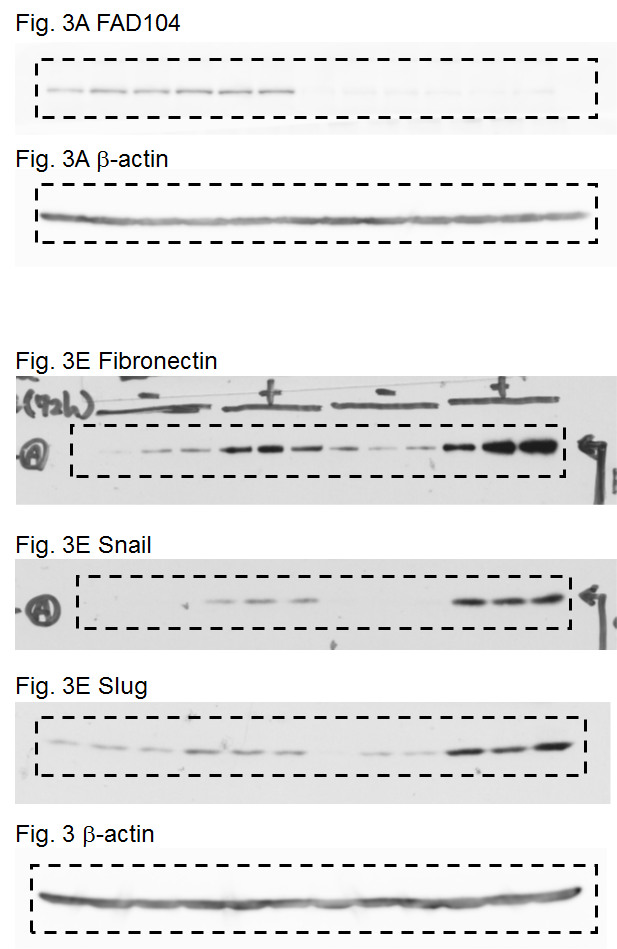


**Supplementary Figure S10. Original uncropped images of blots.**

Boxes highlighted lanes used in Fig. 4.


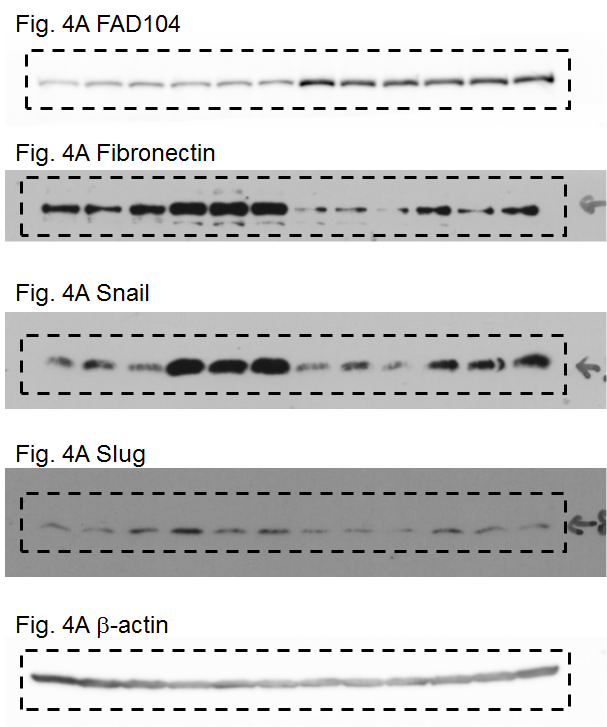


**Supplementary Figure S11. Original uncropped images of blots.**

Boxes highlighted lanes used in Fig. 5.


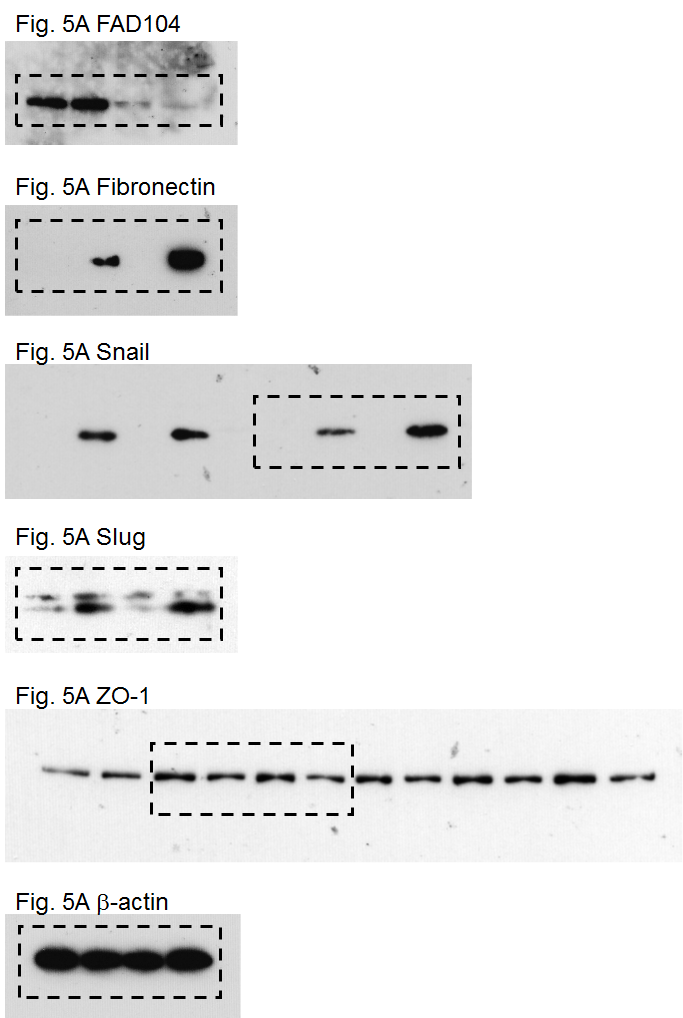


**Supplementary Figure S12. Original uncropped images of blots.**

Boxes highlighted lanes used in Fig. 6.


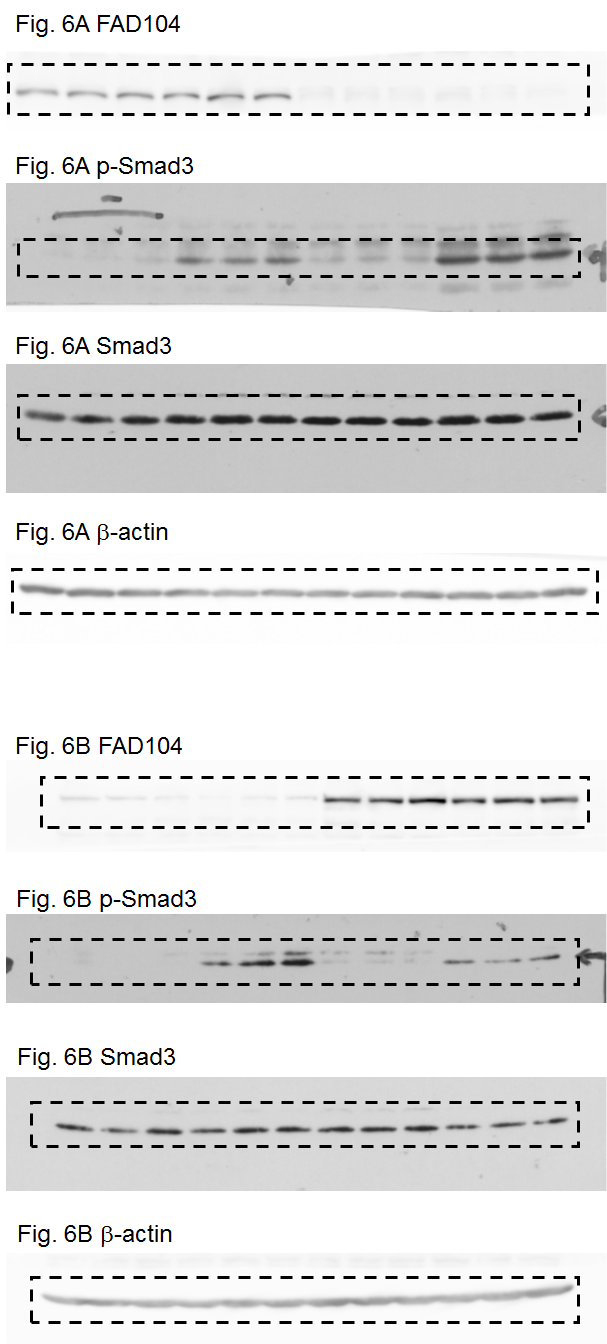


**Supplementary Figure S13. Original uncropped images of blots.**

Boxes highlighted lanes used in Fig. 7.


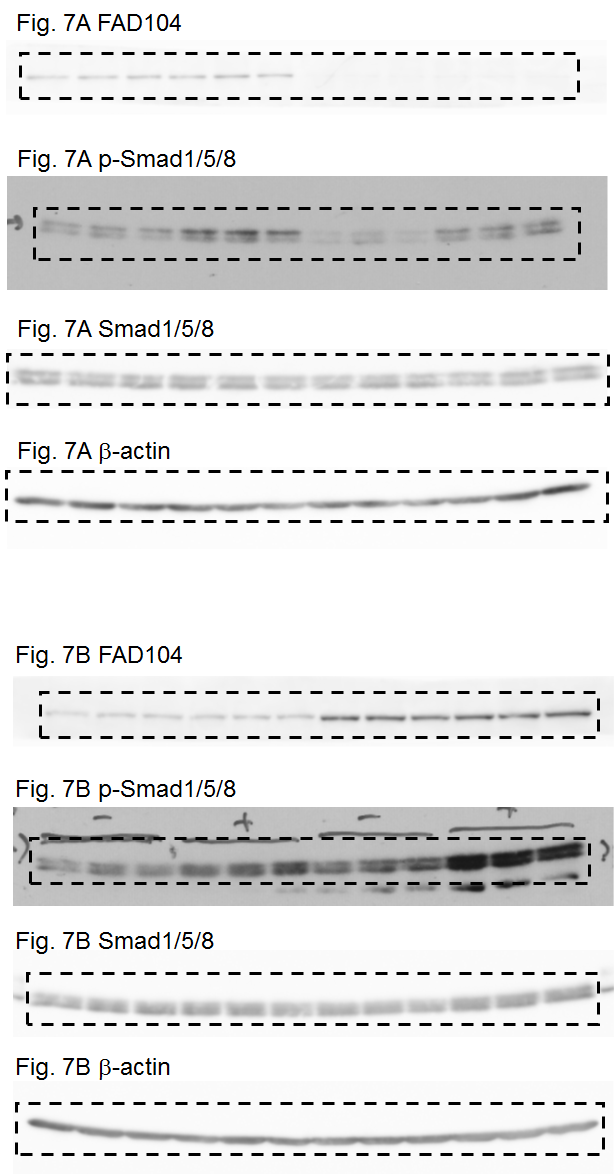


**Supplementary Figure S14. Original uncropped images of blots.**

Boxes highlighted lanes used in Supplementary Figure S1.


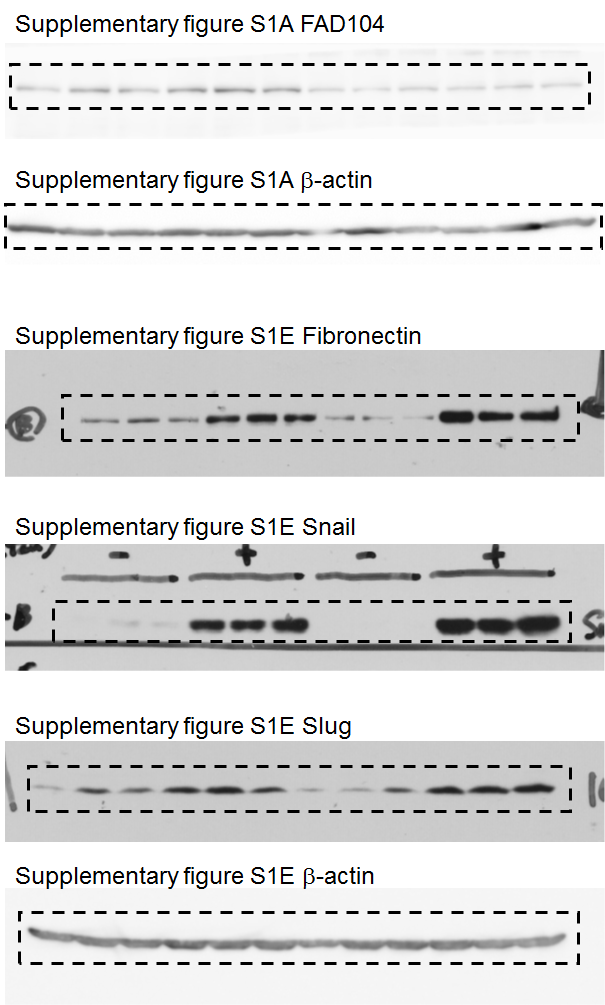


**Supplementary Figure S15. Original uncropped images of blots.**

Boxes highlighted lanes used in Supplementary Figure S2.


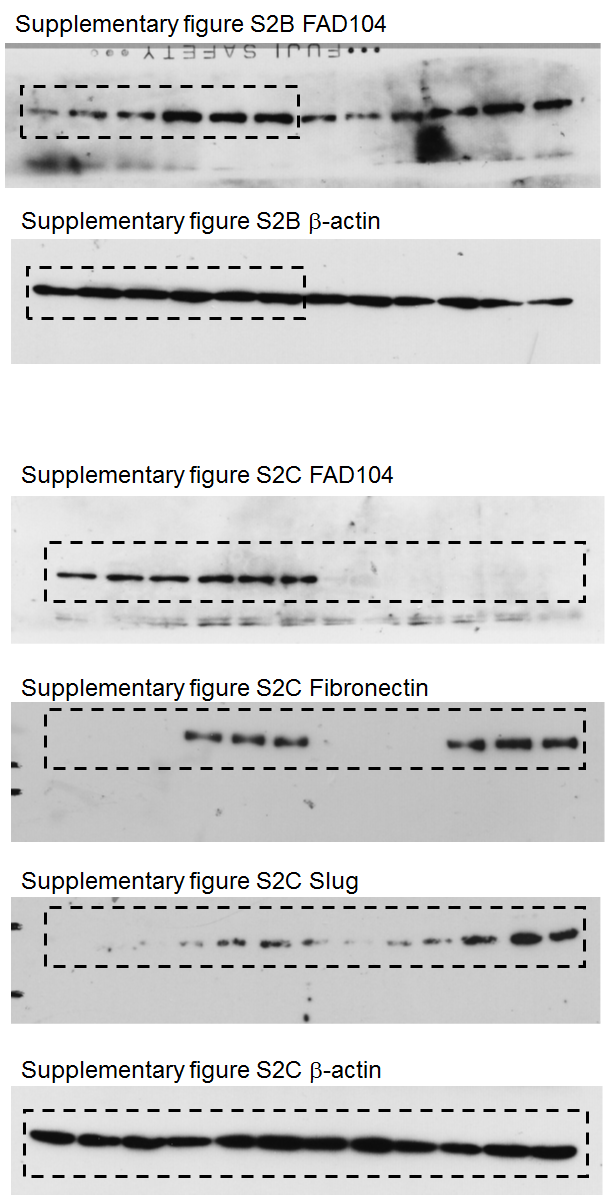


**Supplementary Figure S16. Original uncropped images of blots.**

Boxes highlighted lanes used in Supplementary Figure S3.


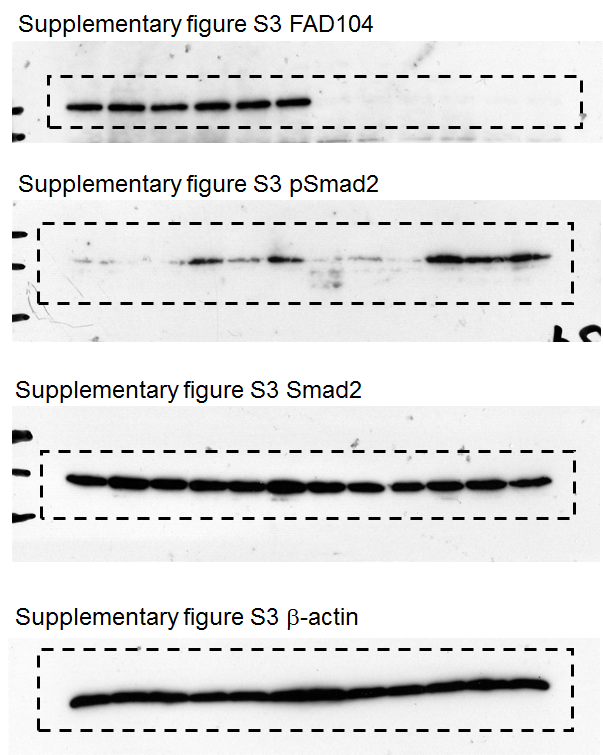


**Supplementary Figure S17. Original uncropped images of blots.**

Boxes highlighted lanes used in Supplementary Figure S4.


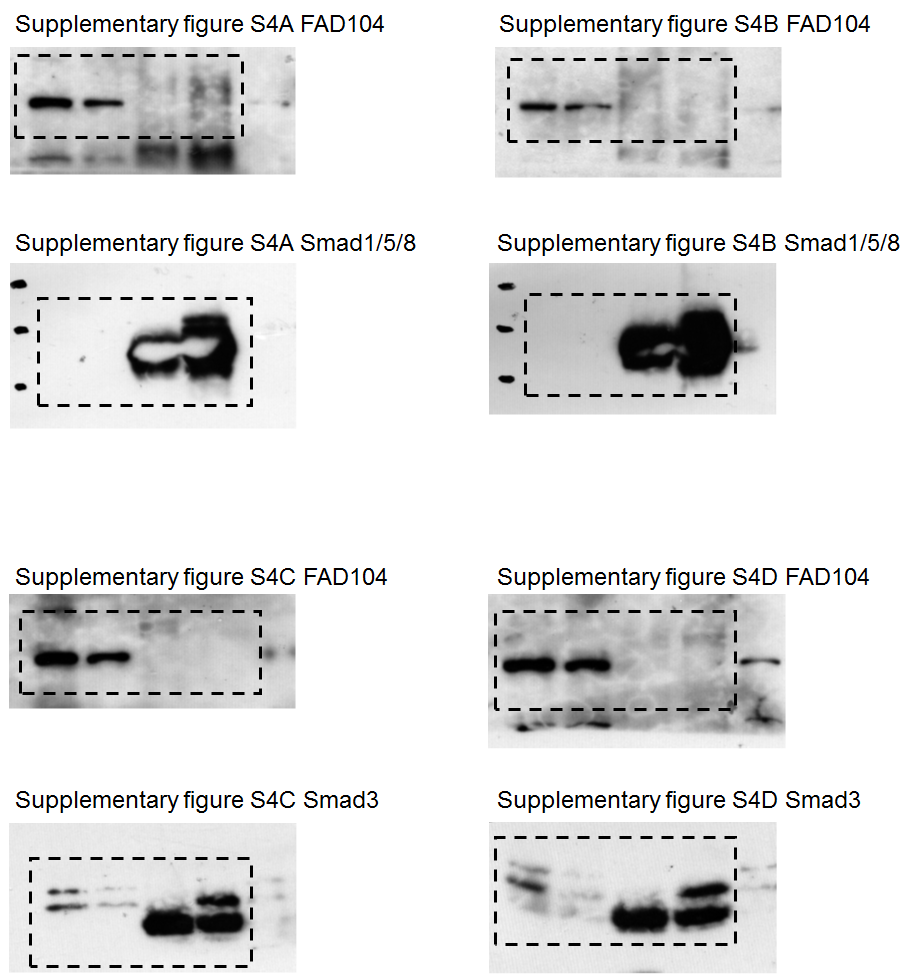


**Supplementary Figure S18. Original uncropped images of blots.**

Boxes highlighted lanes used in Supplementary Figure S5.


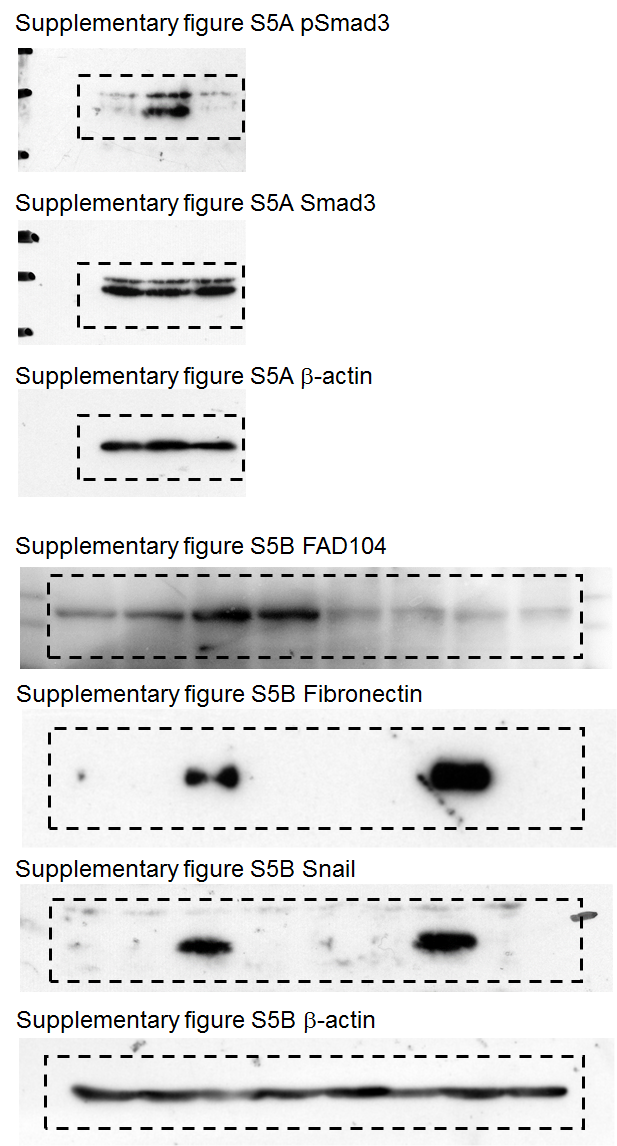


**Supplementary Figure S19. Original uncropped images of blots.**

Boxes highlighted lanes used in Supplementary Figure S6.


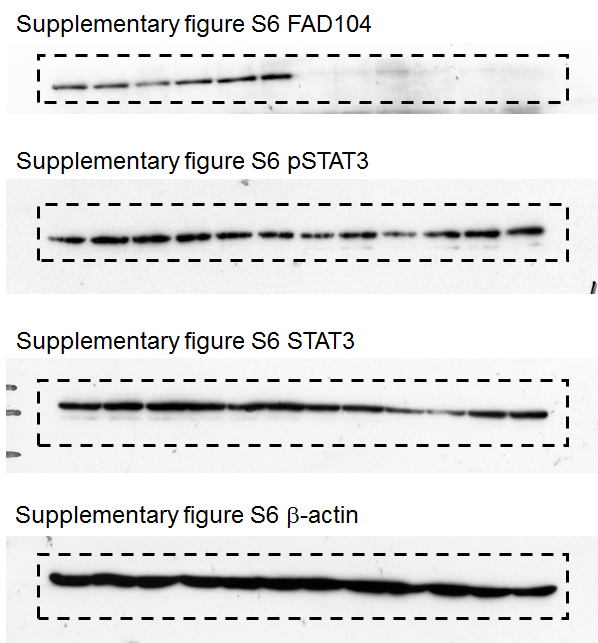

Supplement: Supplementary file 1 — Supplementary Information [file 41598_2017_16555_MOESM1_ESM.doc]
